# Reading Emotions in Faces With and Without Masks Is Relatively Independent of Extended Exposure and Individual Difference Variables

**DOI:** 10.3389/fpsyg.2022.856971

**Published:** 2022-03-17

**Authors:** Claus-Christian Carbon, Marco Jürgen Held, Astrid Schütz

**Affiliations:** ^1^Department of Psychology, University of Bamberg, Bamberg, Germany; ^2^Bamberg Graduate School of Affective and Cognitive Sciences (BaGrACS), Bamberg, Germany

**Keywords:** emotion perception, face mask, personality, emotional intelligence, accuracy, face perception, COVID-19 pandemic, cover

## Abstract

The ability to read emotions in faces helps humans efficiently assess social situations. We tested how this ability is affected by aspects of familiarization with face masks and personality, with a focus on emotional intelligence (measured with an ability test, the MSCEIT, and a self-report scale, the SREIS). To address aspects of the current pandemic situation, we used photos of not only faces *per se* but also of faces that were partially covered with face masks. The sample (*N* = 49), the size of which was determined by an *a priori* power test, was recruited in Germany and consisted of healthy individuals of different ages [*M* = 24.8 (18–64) years]. Participants assessed the emotional expressions displayed by six different faces determined by a 2 (sex) × 3 (age group: young, medium, and old) design. Each person was presented with six different emotional displays (angry, disgusted, fearful, happy, neutral, and sad) with or without a face mask. Accuracy and confidence were lower with masks—in particular for the emotion disgust (very often misinterpreted as anger) but also for happiness, anger, and sadness. When comparing the present data collected in July 2021 with data from a different sample collected in May 2020, when people first started to familiarize themselves with face masks in Western countries during the first wave of the COVID-19 pandemic, we did not detect an improvement in performance. There were no effects of participants’ emotional intelligence, sex, or age regarding their accuracy in assessing emotional states in faces for unmasked or masked faces.

## Significance Statement

The present study validates previous findings that the reading of emotions in faces is impaired when faces are partially covered with a mask (the emotional state of disgust was especially difficult to read)—even 1 year after wearing face masks became common. Although there was a wide range of performance levels, emotional intelligence, assessed with a performance test or with self-reports, did not affect the specific confusion of perceived emotions for faces with or without masks. During a pandemic, it seems necessary to provide and use additional information so that interaction partners’ emotions can be assessed accurately.

## Introduction

A face probably conveys hundreds of dimensions of information, which people can typically read quickly and with little cognitive effort. Besides the socially important dimension of identification, even if we take only a single glance at a person’s face before executing any deeper exploration (Carbon, 2011), their face allows us to assess several other dimensions that are relevant for the raw assessment of a social situation, for example, attractiveness (Carbon et al., 2018), bodyweight (Schneider et al., 2012), and trustworthiness (Willis and Todorov, 2006). The perception of emotions is an additional highly complex ability (Herpertz et al., 2016) as not only basic emotions but even highly differentiated mental states can be inferred from faces, especially on the basis of the region around the eyes (Schmidtmann et al., 2020). All of these pieces of information are assumed to be processed in a rather parallel and highly efficient way (Bruce and Young, 1986), a theoretical claim that indeed has found support from brain research (Haxby et al., 2001). Emotion perception can be considered an aspect of emotional intelligence and is an ability that is related to the wellbeing of both actors and partners (Koydemir and Schütz, 2012) and can be increased through training (Gessler et al., 2021). Such a highly optimized and efficient way of processing facial information can easily be impaired.

During the COVID-19 pandemic, a substantial change in the opportunity to thoroughly perceive facial expressions occurred when the use of face masks became obligatory, which was the case in many countries during the first wave of COVID-19 in May 2020. This global change in the opportunity to perceive facial expressions provides an interesting setting for testing whether the ability to read faces can adapt to such an environmental change. The present study was aimed at analyzing indications of improvements in face reading after having been exposed to partially covered faces for 1 year. For interested readers, we would like to refer to an overview of all kinds of effects documented so far for the use of masks (Pavlova and Sokolov, 2021). However, in the following study, we focus on the effects on emotion reading. We were interested in not only such a possible adaptation but also in variables that could potentially affect the ability to read emotions in faces, foremost the personality variable of *emotional intelligence* (Mayer and Salovey, 1997).

We know from research during the COVID-19 pandemic that adults (Carbon, 2020) as well as (9–11 year old) children (Carbon and Serrano, 2021) are less effective in reading emotions when face masks cover a target’s mouth and nose region. These general findings were replicated several times in 2020 (e.g., Gori et al., 2021; Grundmann et al., 2021) and 2021 (Ramachandra and Longacre, 2022). Specific emotions are especially difficult to discern when face masks are worn. This is the case for all emotions that are strongly expressed by movements in the mouth area (e.g., disgust, anger, sadness, and happiness; see Bombari et al., 2013). For these emotions, recognition is heavily impaired when a face mask is worn (Carbon, 2020). Pre-COVID-19 studies had already shown this general finding, although the results had been inconsistent (see Bassili, 1979; Fischer et al., 2012; Kret and de Gelder, 2012), which calls for further investigation into the specific impairments of face covers for certain emotions.

Emotional intelligence (EI) plays a significant role in the decoding of facial expressions. More precisely, EI is the ability to perceive and regulate emotions in oneself and in others (Mayer and Salovey, 1997). Individuals with better emotion perception skills are especially sensitive to changes in facial expressions and thereby better able to recognize emotions in others (Karle et al., 2018). In their updated Four-Branch Model of Emotional Intelligence, Mayer et al. (2008b) not only make the assumption that individuals high in EI are more adept at recognizing verbal and non-verbal information in others, such as facial or vocal cues, but also differentiate reasoning skills for each of the four branches which range from basic to more complex cognitive processes. According to this model, high EI individuals possess enhanced cognitive abilities that allow them to recognize emotions even under difficult conditions, such as integrating contextual and cultural aspects when decoding emotional expressions. Thus, especially when face masks cover parts of the face, individuals with high emotional intelligence should be better at identifying emotions in others and more confident in their judgments.

Throughout life, individuals continue to develop their emotional intelligence, which includes the ability to perceive emotions (Mayer et al., 2008a), and previous studies have shown that emotional intelligence can be improved by traditional face-to-face training (e.g., Hodzic et al., 2018; Gessler et al., 2021) as well as online training (Köppe et al., 2019), thus highlighting the importance of experience and practice in developing and increasing emotion perception skills. With regard to the current context of a pandemic, individuals who regularly interact with others who wear face masks should be especially skilled at detecting emotions despite the use of face masks. In addition, it is reasonable to assume that individuals have improved their emotion perception skills after having been confronted with partially covered faces for a while. Thus, the respective abilities should be better now than they were at the onset of the COVID-19 pandemic.

When assessing the impact of emotional intelligence, it is important to differentiate between performance-based and self-report measures as performance-based measures are more strongly related to cognitive ability, whereas self-reports are more closely linked to other personality traits (Mayer et al., 2008b; Joseph and Newman, 2010). As a result, studies have revealed only weak correlations between performance-based and self-report measures (e.g., Brackett et al., 2006). For this reason, we employed both performance-based and self-report measures in the present study.

Numerous studies have indicated that individuals’ emotion perception also depends on their attitudes, beliefs, and stereotypes regarding other people. Individuals who did not adhere to wearing face masks in everyday life exhibited more negative attitudes toward face masks (Taylor and Asmundson, 2021) and in the context of the COVID-19 pandemic, individuals who object to the COVID-19 restrictions appear to be separating themselves from the “mainstream” and their previous in-group. As a consequence, individuals who do not wear face masks should be worse at detecting emotions in masked faces (i.e., out-group members).

Last but not least, we were also interested in potential gender differences in processing facial emotions—a topic that has largely been neglected but has piqued interest in recent years, probably influenced by a meta-analysis on this topic in 2013 (Herlitz and Lovén, 2013). The authors of this meta-analysis showed that women had better performance in facial recognition and memories for faces than men (Herlitz and Lovén, 2013) and suspected that this advantage was due to more efficient configural and holistic processing, which also reflects an expertise-based mode of processing (Carbon and Leder, 2005; Rhodes et al., 2006) when processing facial information (e.g., age, Hole and George, 2011). However, in a recent study with a large sample of 343 participants employing the Cambridge Face Memory Test (CFMT; Duchaine and Nakayama, 2006) and the Cambridge Face Perception Test (CFPT; Duchaine et al., 2007), the holistic processing hypothesis was not supported (Østergaard Knudsen et al., 2021). Still, specifically for emotion recognition, it has been shown that women regularly outperform men, for instance, in the acoustic domain (e.g., for the recognition of vocal emotions; Mishra et al., 2019) or the visual domain [e.g., for the recognition of facial emotions (Wingenbach et al., 2018)].

On the basis of these considerations, we tested the following hypotheses, which were previously documented in our preregistration available on the Open Science Framework (OSF), retrievable *via*:^1^

- (H1) Emotion recognition will be better and participants’ confidence in their judgments higher for faces without masks than for faces with masks.
- (H2) Participants’ EI (both performance-based and self-reported) will be positively related to their ability to recognize emotions in faces and to their confidence in doing so.
- (H3) Participants’ emotion recognition will be better and confidence will be higher than emotion recognition in a different but comparable sample measured 1 year ago.
- (H4) Familiarity with face masks will be positively related to emotion recognition and confidence.
- (H5) Positive attitudes toward face masks will be positively related to emotion recognition and confidence.
- (H6) For masked faces, emotion recognition will be worse for emotions in which the mouth area is important than in other emotions.
- (H7) Women will show better emotion recognition and higher confidence than men.
- (H8) Younger participants will show better emotion recognition and higher confidence than older adults.

## Experimental Study

The major aims of the present study were to gain knowledge about whether the ability to process facial emotions is adaptive and can be affected by presentation (masks vs. no masks), time (during the COVID-19 pandemic), and participants’ sex and age. Further, we aimed to test whether performance in the processing of facial emotions can be affected by participants’ emotional intelligence (EI) or participants’ attitude toward face masks.

## Materials and Methods

### Participants

#### Calculation of the Sample Size

We calculated the required sample size N using a test model that included EI as a fixed factor (Model 2) compared with a baseline model without EI (Model 1). As we followed a test strategy based on linear mixed models (LMMs), we calculated the test power a priori using the R package simr (Green and MacLeod, 2016). To compare the two models, we set the intercept to 60% (the dependent variable was the performance level, which could range from 0 to 100%, so 60% indicates a medium-high performance level given a six Alternative Forced Choice (AFC) design with a 16.7% base rate). The slopes of the fixed effects of emotions were set to −2, +5, +5, +5, and + 10 for the different emotional states (disgust, anger, neutral, happiness, and fear, respectively) compared with the emotional state of sadness, based on typical findings for these emotions (e.g., Carbon, 2020). For Model 2, we set the slope of the fixed effect of EI to +2, the random intercept variance was set to 10, and the residual standard deviation was set to 20. With 1,000 simulations, we obtained a test power of 90% [95% CI (89.16, 92.79)] with a sample size of *N* = 46.

We recruited the *N* we needed plus 5 additional individuals (initially we planned to oversample up to *N* = 54) given that invalid data are typically expected from about 1/5 of participants, but a preliminary inspection of the data (looking for potential indicators of data that should be excluded as documented in the preregistration, i.e., very low performance and many missing data points) indicated a much smaller amount of invalid data; only two participants were excluded due to the preregistered outlier criterion of having correctly identified the emotional states of faces without face masks in less than 50% of the cases.

#### Sample

The final sample consisted of 49 participants [*M*_age_ = 24.8 years (18–64 years), *N*_female_ = 39], yielding a *post-hoc* power of 92.10% [95% CI (90.25, 93.70)]. People had been recruited by different online advertisements; they were not directly incentivized but had the option to participate in a lottery with prizes ranging from 10 to 100 Euros (5 × 10 Euros, 1 × 50 Euros, and 1 × 100 Euros).

### Material

**Figure 1:**
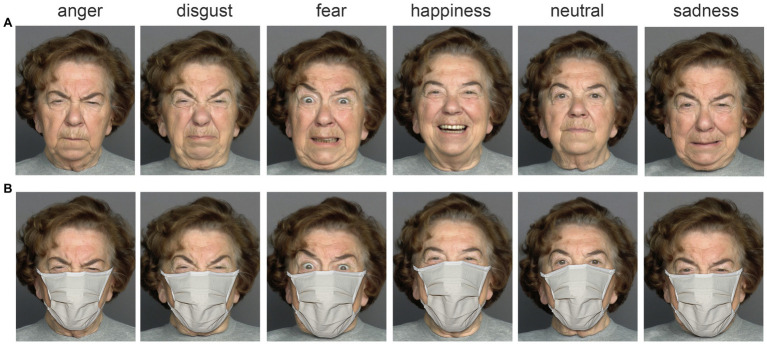
The figure illustrates the six emotional variations (anger, disgust, fear, happiness, neutral, and sadness) of one person without **(A)** and with **(B)** a face mask. This specific person was not part of our experimental material but is presented here for illustrative purposes. The authors would like to thank the Max Planck Institute for providing the baseline stimuli (without masks), which came from the MPI FACES database (Ebner et al., 2010).

The baseline face stimuli without masks were obtained from the MPI FACES database (Ebner et al., 2010) on the basis of a study-specific contract effective 19 April 2021. We used frontal photos of six Caucasians (three female and three male) who belonged to three different face age groups [*young*, *medium* = middle-aged, *elderly* with average perceived ages of 25.5, 41.5, and 67.0 years, respectively, as shown in a previous study by Ebner et al. (2010)] as baseline images to which we subsequently applied face masks with a graphics editor. Each person showed the emotional states *anger, disgust, fear, happiness*, and *sadness*, and one *neutral expression*. Each *face sex* × *face age group* cell was represented by one specific person. We doubled all of the 2 [face sex] × 3 [face age groups] × 6 [emotional states] = 36 baseline pictures to apply a typical face mask used during the COVID-19 pandemic (a so-called “community mask” colored beige). For each manipulated picture, the mask was individually adapted to fit the different faces perfectly; we added realistic shadow effects to further increase the realism of the pictures with face masks (Figure 1).

Overall, the material consisted of 2 [no mask vs. mask] × 36 = 72 facial stimuli, half of the original material originally used by Carbon (2020). Specifically, we used only one of the two face age group representatives per sex from the original study. This was done to reduce the total duration of the present study, which was substantially extended by adding the personality variables.

### Apparatus

#### Study Platform

As the study platform, we used the online tool SoSci Survey,^2^ which is freely available for non-profit-oriented scientific projects.

### Measures

#### Ability-Based Emotional Intelligence

We used the faces and images subtasks from the German version of the Mayer-Salovey-Caruso Emotional Intelligence Test (MSCEIT; Steinmayr et al., 2011) to assess ability-based emotion perception. Participants used a 5-point scale to indicate the degree to which each of five emotions was expressed in a photograph (faces subtask) or pictures of landscapes and abstract patterns (images subtask). In line with previous research, internal consistency analyses revealed a Cronbach’s alpha of *α* = 0.683 for the faces subtask, *α* = 0.833 for the images subtask, and *α* = 0.857 for the emotion perception scale in this study.

#### Self-Reported Emotional Intelligence

The Perceiving Emotion subscale from the German version of the Self-Rated Emotional Intelligence Questionnaire (SREIS, see Vöhringer et al., 2020) was employed to assess emotion perception skills *via* self-report. Participants rated their emotion perception skills on a 5-point scale ranging from 1 (*very inaccurate*) to 5 (*very accurate*). Again, internal consistency analyses were computed, and Cronbach’s alpha for the SREIS was *α* = 0.520.

#### Attitudes Toward Face Masks

Participants’ overall attitude toward face masks was measured with a single item “What is your personal opinion toward the mandatory use of masks?” with the response options: “I do not consider the mandatory use of masks a problem,” “To me the mandatory use of masks is annoying but bearable,” and “I consider the mandatory use of masks unreasonable and burdensome.” Further, we employed the 12-item scale developed by Taylor and Asmundson (2021) with answers that were rated on a 7-point scale ranging from 1 (*strongly disagree*) to 7 (*strongly agree*) to allow for a more fine-grained analysis of face mask attitudes. Internal consistency was *α* = 0.906 for the 12-item scale.

#### Face Mask Use

Participants indicated their individual face mask use (FamiliarityOwnMask) by rating the item “On average, how many hours a day do you wear a face mask?” on a 10-point scale ranging from 1 (*max. 30 min*) to 10 (*more than 8 h*). In addition, we asked participants to rate their daily duration of interpersonal contact with face masks (FamiliarityOthersMasks) by answering the item “How many hours per day are you in face-to-face contact with others who wear a face mask?” on a 10-point scale ranging from 1 (*max. 30 min*) to 10 (*more than 8 h*).

#### Procedure

We conducted the study between 13 July 2021 (13:09 local time; CEST) and 19 July 2021 (11:52 local time; CEST) during the end of the third wave of the COVID-19 pandemic in Germany. Each participant gave written consent to participate in the study; data were collected anonymously. We first asked general demographic questions about participants’ age and sex. Then, the experimental part began. We fully randomized the order of the stimuli for each participant. The participant’s task was to assess the depicted person’s emotional state using a six Alternate Forced Choice (AFC) task where all six of the possible emotional states were shown as written labels (in German: *anger*, *disgust*, *fear*, *happiness*, *neutral*, and *sadness*) along with a confidence scale. So by clicking on one scale point of the respective confidence scale the participants indicated the perceived emotional state as well as the confidence with just one click. The confidence scale was used to assess the participant’s confidence in their recognition of the respective emotion expression on a 7-point scale ranging from 1 (*not confident at all*) to 7 (*very confident*). We did not set a time limit for the assessment but asked participants to respond spontaneously. The next trial started after the participant pushed a response key, initiated by a short, intermediate pause with a blank screen presented for half a second. After the experimental part, we administered all questionnaires and single questions. Participants took 27.5 min on average to successfully complete the whole study. We obtained ethical approval for the general psychophysical study procedure from the local ethics committee of the University of Bamberg (Ethikrat).

## Results

We employed R 4.1.2 (R Core Team, 2021) to process and analyze the empirical data, mainly by using linear mixed models (LMM) in the package *lme4* (Bates et al., 2015). The preregistered study as well as the (anonymized) data can be found on the OSF.^3^

**Figure 2:**
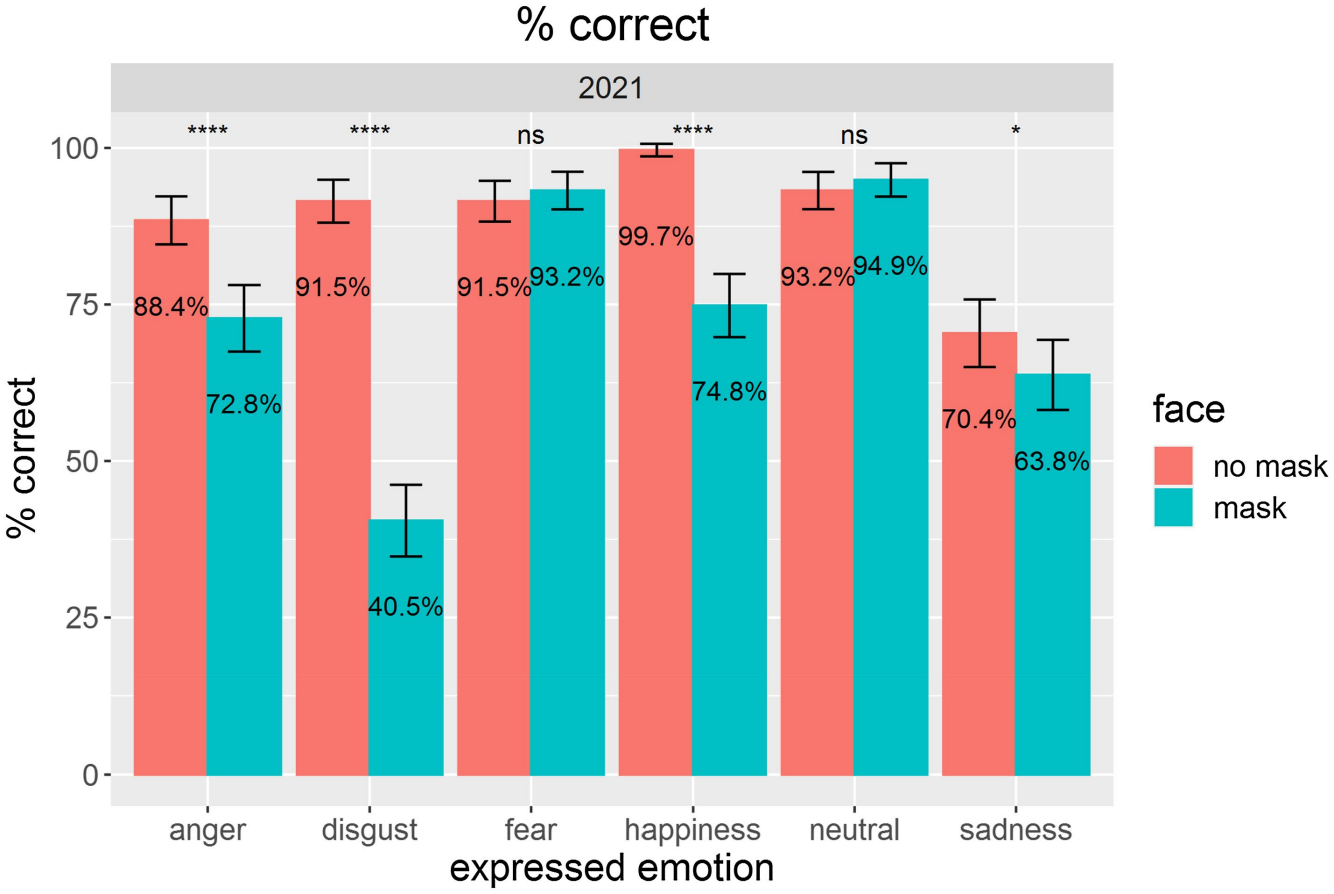
The figure demonstrates mean performance levels for assessments of emotional states for faces without masks (red) compared with faces with masks (blue). Error bars indicate 95% confidence intervals (CIs) according to Morey (2008). Pairwise comparisons of the presentation conditions were calculated *via* undirected paired t-tests. ^*^p < 0.05. ^****^p < 0.0001. Nonsignificant results are marked with ns.

Performance was calculated as a percentage of correct data, confidence was converted to percentage ratings such that the minimum confidence rating of 1 corresponded to 0%, and the maximum confidence rating of 7 corresponded to 100% confidence. We obtained a mean performance level for the baseline condition of faces without masks of *M* = 89.1%, which is remarkable given that a chance rating for a six AFC is 16.7%. For the faces with masks, the performance level dropped to 73.3%, which was still much higher than chance. The drop in performance was evident for four of the six emotional states from the visual inspection of Figure 2—for anger, disgust, happiness, and sadness.

**Figure 3:**
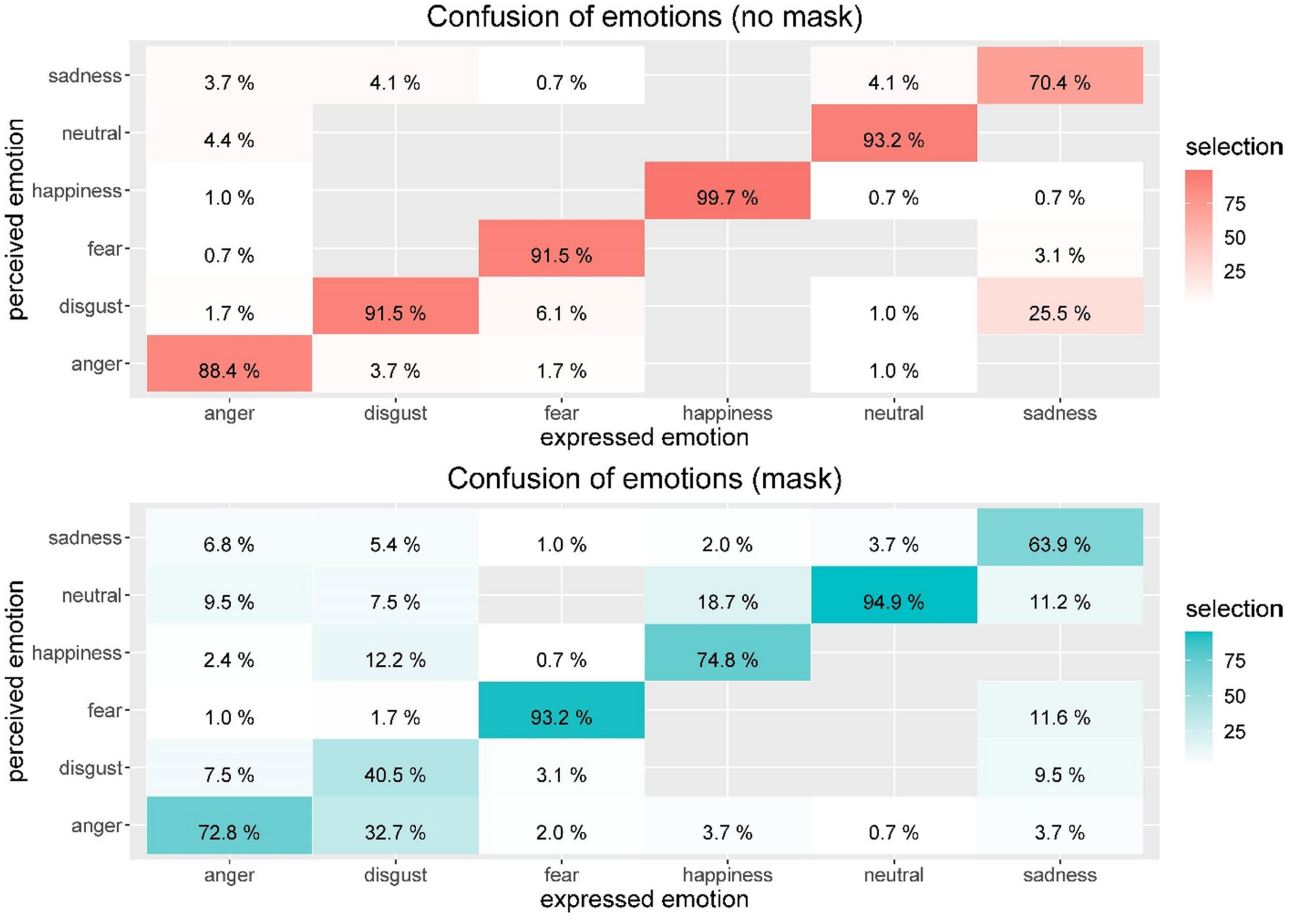
This figure shows the confusion matrices for expressed versus perceived emotions for the original faces without face masks (top red) and faces with face masks (bottom blue). Mean performance levels in assessing the emotional states are given by percentage correctness rates (if >0.5%, otherwise data were suppressed for better readability of the matrices). The better the performance, the more saturated were the confusion matrix cells.

We inspected the drop in performance when faces wearing masks had to be inspected by observing the confusion matrices for expressed versus assessed emotions. As Figure 3 indicates, there was confusion of emotions even when the entire face (without a mask) was shown. This was especially true for sadness, which was correctly identified in 70.4% of the cases and misinterpreted as disgust in about 25% of the cases. Recognition of the other emotions was quite good with correctness levels above 88.4% (for anger) or higher (e.g., 99.7% for happiness).

When faces were shown with masks, participants were more confused about which emotion was displayed. This was particularly the case for disgust, which participants very often misinterpreted as anger (32.7%). Sadness was diffusely assessed, with no clear misinterpretation for a single emotion, but with a broad spectrum of interpretations ranging across fear, neutral, disgust, or anger. The exceptions to the rule were neutral and fear, which were not negatively affected by adding a face mask.

**Figure 4:**
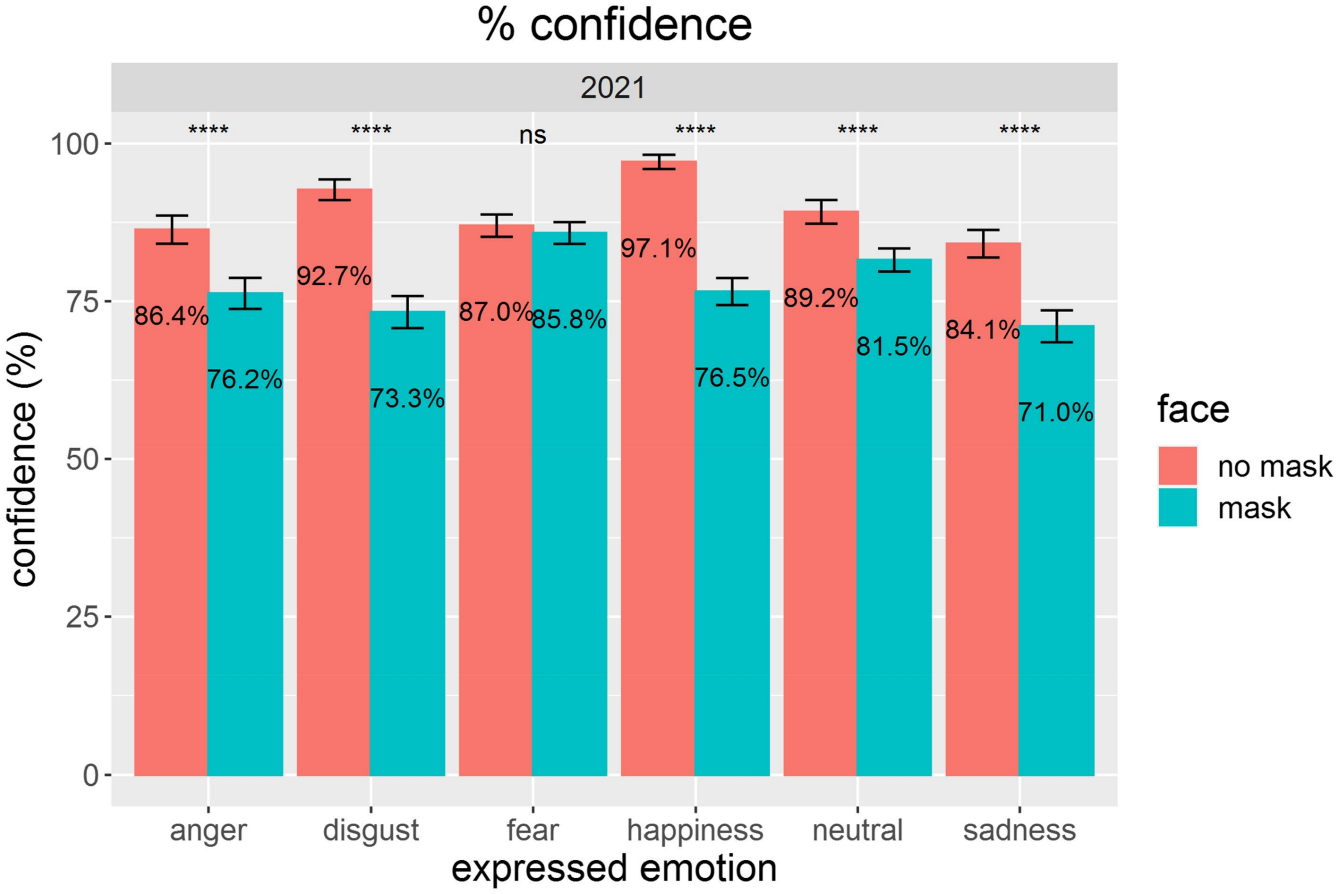
This figure shows mean confidence levels for assessments of emotional states for faces without masks (red) compared with faces with masks (blue). Error bars indicate 95% CIs according to Morey (2008). Pairwise comparisons of the presentation conditions were calculated *via* paired t-tests. ^****^p < 0.0001. Nonsignificant results are marked with ns.

We also analyzed the data on participants’ confidence in choosing the respective emotional state. As Figure 4 indicates, participants showed numerically lower confidence when assessing masked faces. With five out of six emotions, we found statistically significant drops in confidence: for anger, disgust, happiness, neutral, and sadness.

We tested the effect of wearing masks on performance and confidence with two separate linear mixed models (LMMs). As the null model (Model 0), we used a simple one containing the participants and baseline stimuli as random intercepts and *facial emotion* as a fixed effect. For Model 1, we added *face mask* (face with a mask vs. without a mask) as a fixed factor. The coefficient of determination for each model was calculated *via* a likelihood-ratio test utilizing the R package *MuMIn* (Barton, 2019).

For both dependent variables (i.e., performance and confidence), we obtained significant effects of *face mask*, *p*s < 0.0001, with a drop in performance of 15.8% and a drop in confidence of 11.9%. This result supported H1. For details, see Table 1.

**Table 1:** Comparison of different linear mixed effects models.

Dependent variable/tested model	***df***	AIC	logLik	Cond.*R*^2^	Against	*p*(*χ*^2^)
**%correct**						
#0: null	9	35,483	−17,732	0.128		
#1: + Mask	10	35,316	−17,648	0.168	#0	<0.0001
#2: + EI + SREIS	12	35,320	−17,648	0.168	#1	0.9081 *n.s*.
#3a: + FamiliarityOthers	11	30,243	−15,111		#1	<0.0001
#3b: + FamiliarityOwn	11	35,318	−17,648	0.168	#1	0.8360 *n.s*.
#4: + attitudeMasks	11	35,318	−17,648	0.168	#1	0.5975 *n.s*.
#5: + exprEmo:Mask	15	35,177	−17,527	0.224	#1	<0.0001
**%confidence**						
#0: null	9	37,253	−15,317	0.240		
#1: + Mask	10	30,246	−15,113	0.324	#0	<0.0001
#2: + EI + SREIS	12	30,324	−15,113	0.324	#1	0.9468 *n.s*.
#3a: + FamiliarityOthers	11	30,243	−15,111	0.324	#1	0.0325
#3a: + FamiliarityOwn	11	30,245	−15,111	0.324	#1	0.0838 *n.s*.
#4: + attitudeMasks	11	30,247	−15,113	0.324	#1	0.5115 *n.s*.

*The table shows the results of linear mixed effects analysis of different models in comparison with less complex models, separated by the two tested dependent variables % correct (percentage of correct emotion classifications) and % confidence (for correct emotion classifications). FS, fixed slopes (fixed factors); RS, random slopes (random factors); df, degrees of freedom; *R*^2^, conditional coefficient of determination, based on the likelihood-ratio test; and “against” indicates the model against which the current model was tested, *p*(*χ*^2^) provides the probability of accepting a significant effect despite a nonexistent difference regarding the more complex model versus the model specified in the “against” column*.

We also tested H2, in which we focused on the relationship between participants’ ability-based or self-reported emotional intelligence (EI) and their performance and confidence in assessing emotional expressions in faces. We used an LMM approach with Model 2 including EI (ability-based emotional intelligence) and SREIS (self-reported emotional intelligence) as fixed factors compared with Model 1 where these EI-related scores were not included. We also analyzed the correlation between EI and SREIS, which turned out to be nonsignificant, *r* = 0.01, *p* = 0.93, *ns*. For both dependent variables, we did not explain more variance by including EI-related scores (see Table 1). Thus, H2was not supported.

We also tested H3, which proposed that people in the present sample from 2021 would have higher scores (higher performance and higher confidence, respectively) than the original sample tested with the same experimental procedure in 2020. Note: As the 2020 study used twice as many stimuli, we analyzed only the material used in both studies. Again, we followed an LMM approach, this time with the merged data set, which comprised the 2020 sample consisting of 41 participants and the 2021 sample consisting of 49 participants, yielding a total of *N* = 90 participants. This time, as the null model, we used Model A0, which in fact reflected the previous Model 0 but was fed by the overall data set comprising the 2020 and 2021 data. Model A1, which included *Study* (2021 vs. 2020) as a fixed factor, was not able to explain additional variance for the performance or the confidence data. Thus, H3 was not supported, *p*s > 0.7638.

Regarding H4, we tested whether greater familiarity with face masks would lead to better performance or confidence, respectively, in assessing facial emotions. This was done with Model 3 to which we added *familiarity*. We measured the familiarity with face masks in two ways: The first item asked about familiarity with face masks in terms of a person’s own use of face masks per day (*FamiliarityOwnMask*), whereas the second item asked about familiarity with face masks in terms of perceiving other people with face masks (*FamiliarityOthersMasks*). As the two aspects capture different perspectives of the aspect of familiarity, we decided to add them to Model 3 as two different fixed factors (Models 3a and 3b, respectively), which we tested against Model 1. We revealed that *FamiliarityOthersMasks* was significantly related to higher performance as well as higher confidence in the assessment of facial emotions, whereas *FamiliarityOwnMask* failed to reach significance with the given power.

We tested H5, which were about the relationships between people’s attitudes toward face masks and the dependent variables performance and confidence, respectively, in assessing facial emotions. Again, we tested this against Model 1 with an LMM. Model 4 which included the additional fixed factor *attitudeMasks* did not explain more variance than Model 1, so H5 was rejected.

Regarding H6, we analyzed the selective decrease in performance in assessing certain facial emotions when faces were shown with masks, again utilizing an LMM approach. We did not use *face mask* as a fixed factor as in Model 1 but as an interactive effect with *exprEmo* and tested this Model 5 against Model 1. As expected, we found a stronger effect of face masks on performance in identifying facial emotions for which the mouth area was indicative (anger, disgust, happiness, and sadness) versus nonindicative (fear). As shown in Table 2, we obtained a nonsignificant effect of the interaction between *face mask* and the emotion *fear*, probably because fear is mainly expressed by the eyes. By contrast, we obtained clearly reduced performances in detecting anger, disgust, happiness, and sadness when a mask covered the mouth region. The largest effect was observed for disgust.

**Table 2:** Results of the linear mixed effects analysis for emotion recognition performance testing Model 5 against Model 1.

Predictors	Estimates	*p*	*df*
(Intercept)	93.20 ^***^	**<0.001**	3,514.00
Neutral	*Reference*		
Anger	−4.76	0.094	3,514.00
Disgust	−1.70	0.549	3,514.00
Fear	−1.70	0.549	3,514.00
Happiness	6.46 ^*^	**0.023**	3,514.00
Sadness	−22.79 ^***^	**<0.001**	3,514.00
exprEmo_anger:Mask	−17.35 ^***^	**<0.001**	3,514.00
exprEmo_disgust:Mask	−52.72 ^***^	**<0.001**	3,514.00
exprEmo_fear:Mask	−0.00	1.000	3,514.00
exprEmo_happiness:Mask	−26.53 ^***^	**<0.001**	3,514.00
exprEmo_sadness:Mask	−8.40 ^*^	**0.037**	3,514.00
No mask	*Reference*		
Mask	1.70	0.549	3,514.00
ICC	0.05		
*N*_depictPers_	6		
*N*_CaseID_	49		
Observations	3,529		
Marginal *R*^2^/Conditional *R*^2^	0.179/0.224		
AIC	3,5084.229		
Log-likelihood	−17,527.114		

>The table shows the statistics for all involved fixed effects in the linear mixed effects analysis for Model 5, regarding the tested dependent variable % correct (percentage of correct emotion classifications). Abbreviated notations for the terms were used to save space: exprEmo_XY = facial emotion, e.g., anger; Mask = face with face mask. Significant values of *p* are in bold. ^*^p < 0.05 and ^***^p < 0.001.

H7 addressed effects of participants’ sex on performance and confidence, respectively, of correctly assessing the emotional states depicted in faces. We tested both hypotheses with an LMM by adding the fixed factor of participants’ sex (Model 6) against Model 1. There was no significant effect of participants’ sex for performance or for confidence, *p*s > 0.5970.

H8 tested effects of participants’ age on performance and confidence. We tested both hypotheses with an LMM by adding the fixed factor of participants’ age (Model 7) against Model 1. There was no significant effect of participants’ age for performance or for confidence, *p*s > 0.1121.

## Discussion

During the different waves of the COVID-19 pandemic, face masks have consistently been used as simple, cheap, and easy-to-apply methods to effectively reduce the transmission of CoV-SARS2 (Howard et al., 2021). Having started with low acceptance in Western countries due to the lack of familiarity with its use in early 2020, the face mask became an ideogram of the pandemic, and with everyday experience, acceptance increased (Carbon, 2021).

In the present study, we tested how individual difference variables were related to the ability to assess emotions in faces with and without masks and whether exposure to masks has improved the ability to infer emotional states from the remaining facial area that is not covered by the mask. We know from the literature that such little facial information is sufficient for recognizing mental states, such as being confident, doubtful, upset, or uneasy (Schmidtmann et al., 2020). This is astonishing because, in typical everyday life situations, aside from a pandemic such as the COVID-19 pandemic, we are typically not exposed to such a reduction in facial information. When we conducted the present study at a time when people in Germany had been obliged to wear face masks in public for more than 1 ¼ years. This led us to assume that people would be familiar with face masks and skilled in reading emotions in partly covered faces. Despite this high level of familiarity with face masks, we observed reduced performance and confidence when people interpreted masked faces. Moreover, people were not better than people had been a year earlier (in April 2020, see also Mitze et al., 2020), but we have to be very cautious about making this comparison because we did not test the same people nor did we use a matched sample. Still, the key parameters were very similar (German sample, mean age differed by only 1.8 years, comparable relative number of female to male participants).

Confusion between emotions in 2021 was similar to the effects documented 1 year earlier: whereas neutral faces and fear were usually well detected even when a face mask was present, anger was often misinterpreted as neutral, disgust, or sadness. Furthermore, sadness was often misinterpreted as neutral, fear, or disgust. Most dramatically, disgust was misinterpreted in nearly 1/3 of the cases and was identified as anger, happiness, or a neutral expression. Interestingly, happiness was often misinterpreted as a neutral expression. Such misinterpretations could be socially relevant in everyday life. For instance, if our counterpart signals affirmation or gratitude by expressing a happy face, we might not see this positive feedback and could misinterpret this social situation. Familiarity with masks was not a relevant factor regarding the ability to read faces either: Only when people were very often exposed to masked faces was their confidence slightly higher.

When analyzing the specific drops in performance or confidence regarding the recognition of emotions in masked faces, we found the expected result—that all emotions that are strongly expressed by the mouth area (e.g., the “smiling mouth” for happy faces or drawing down the labial angles for sad faces) were particularly impaired when a mask covered the mouth area.

In the present study, we further addressed the question of whether emotional intelligence (EI) is linked to the ability to assess facial emotions (with and without masks). However, neither ability-based nor self-reported EI was significantly linked to performance or confidence ratings. Due to the low internal consistency of the SREIS in this sample, the respective results should be interpreted with caution. We also did not find a relationship between attitude toward wearing masks and ability or confidence—so even people who had negative attitudes about using face masks were not worse at or less sure about identifying emotions.

Most of our effects were based on confidence as the dependent variable. When analyzing who was specifically affected by face masks, we found that people who were high performers in the condition without masks were more affected than others. However, this was true only for the confidence ratings but not for actual performance. We did not find an effect of participants’ sex on performance or confidence. Similarly, age had no effect.

Taken together, we were able to detect clear impairments in the ability to read facial emotions as soon as a face was partly covered by a face mask. With the exception of disgust, where we found a dramatic reduction in performance and confidence, most people were less impaired than one might think, considering how much the faces were covered. With an average performance level of 73.3%, participants were much better than chance, a level that had similarly been observed in children only recently (Carbon and Serrano, 2021).

It is important to consider that we used high-quality face stimuli, which had been tested for clear emotional expressions and were characterized by perfect illumination and a frontal perspective. Moreover, participants were able to look at the pictures without time pressure and with the opportunity to fixate perfectly. Such ideal presentations are not available in everyday life, where faces have to be read in complex situations (Yang and Huang, 1994) and where time to inspect the counterpart is limited because of other task requirements or cultural factors, such as maximally accepted eye fixation durations (Haensel et al., 2021). In other words, in everyday life, the general performance of recognizing emotions is probably much lower, and facial masks would be an additional burden. We do not really know how much we gain, on the other side, when encountering faces in reality, e.g., by using dynamic 3D information (see Dobs et al., 2018).

Still, does such reduced information jeopardize communication? Basing the understanding of our counterpart’s emotional or mental state on only facial expressions would be pretty inefficient. More than this, reliance on just one source of information would be reckless and improbable from an evolutionary point of view. Typically, highly developed social species such as humans use different channels of sensory inputs (Shi and Mueller, 2013) and build mental models to predict plausible outcomes (Johnson-Laird, 2010). Furthermore, humans can disambiguate difficult situations (e.g., the uncertain status of a counterpart) by verbally posing questions or simply by waiting for additional information.

On the basis of a comparison of data from 2020 to 2021, we showed that people apparently did not easily adapt their emotion reading skills but people can use additional sources of information. We only tested the loss of information in one channel, but other researchers collected supplemental information (for a short list of ways to cope with the loss of information, see Mheidly et al., 2020).

As a conclusion we speculate that the first important step toward facilitating communication among people who wear face masks would be to raise awareness regarding the challenges to communication that hail from the loss of facial information. Additional steps are to utilize information on body language and gestures. Considering the social situation, we are currently in, we can also use scripts and schemata typically employed in such situations, which help us predict what others will feel and how they will be affected by the current situation. Lastly, we can directly approach our counterparts and explicitly ask them whether pieces of information are missing.

The COVID-19 pandemic comes as a worldwide crisis with specific challenges. The impaired ability to read facial information is definitely one of these challenges. However, intelligent species adapt adequately to better cope with such a situation by developing new means of communication and social interaction. In the end, true social competence manifests itself in the ability to adapt to given task demands. If we use this ability flexibly, we will effectively cope with the communicative challenges inherent in the present pandemic.

## Data Availability Statement

The datasets presented in this study can be found in online repositories. The names of the repository/repositories and accession number(s) can be found at: https://osf.io/rfmv7.

## Ethics Statement

We obtained ethical approval for the general psychophysical study procedure from the local ethics committee of the University of Bamberg (Ethikrat). The participants provided their written informed consent to participate in this study.

## Author Contributions

C-CC and AS had the initial idea for this research. C-CC made the statistics and the figures and wrote the initial paper. MH enriched the statistics by adding consistency scores. AS and MH revised the manuscript. All authors contributed to the article and approved the submitted version.

## Funding

The publication of this article was supported by the OpenAccess publication fund of the University of Bamberg.

## Conflict of Interest

The authors declare that the research was conducted in the absence of any commercial or financial relationships that could be construed as a potential conflict of interest.

## Publisher’s Note

All claims expressed in this article are solely those of the authors and do not necessarily represent those of their affiliated organizations, or those of the publisher, the editors and the reviewers. Any product that may be evaluated in this article, or claim that may be made by its manufacturer, is not guaranteed or endorsed by the publisher.

## Footnotes

^1^ https://osf.io/c8zmn/

^2^ https://www.soscisurvey.de/

^3^ https://osf.io/rfmv7
